# The Report of the Unofficial Commission of Investigation

**Published:** 1917-03-17

**Authors:** 


### NATIONAL INSURANCE ACT DEVELOPMENTS.

### The Report of the Unofficial Commission of Investigation.

Following rapidly upon the publication of the Final Report of the Departmental Committee of Inquiry appointed by the Treasury, there has now appeared the Report of the Commission of Investigation appointed by the Faculty of Insurance. It is a publication which cannot be ignored, but from some points of view its existence is to be deprecated. Although entirely unofficial, the Commission included among its members such prominent Parliamentarians as Mr. John Hodge (the original chairman), Mr. R. E. Prothero, and Mr. J. W. Pratt—each of whom resigned on accepting office in the present Government—Mr. G. W. Currie (the succeeding chairman), Mr. Handel Booth, and Sir J. D. Rees, besides other well-known men of affairs. Its composition therefore gave it considerable weight, added to which the fact that many of its sittings were held in a committee room of the House of Commons imparted a further semblance of authority. In these circumstances it was natural that many witnesses were found ready to give evidence, and their evidence was reported at some length in the newspapers at the time the sittings were held. It may be doubted, however, whether this evidence was really representative or entirely trustworthy. Owing to the unofficial character of the investigation the largest and most important approved societies declined to give evidence, whilst some of the witnesses preferred, so far at least as the publication of their evidence was concerned, to remain anonymous.

The investigation certainly had one advantage denied to the Official Inquiry—namely, that it was not hampered by restricted terms of reference. The limitation imposed upon the Departmental Committee necessitated their confining their attention to the operations of approved societies—although even here there was plenty of scope for their labours, both with regard to the fundamental financial basis and the simplification of the administration of sickness and disablement benefits. But the Commission of Investigation was able to extend its view to include in addition the administration of medical and sanatorium benefits. On these extra subjects there is no doubt that the evidence they obtained will be useful when amending legislation is proposed, and more particularly as it was made clear that the treatment of tuberculosis under the dual control of the National Health Insurance Authorities and the Local Government Board is not in all respects satisfactory.

### Bias against Official Report.

But the Commission started its operations with a bias, and that bias was in opposition to the Official Departmental Committee. It was, in fact, due to the disagreement of a single member of the Committee—Mr. P. Rockliffe—from the remainder of his colleagues on that body that the Commission owed its inception. That disagreement arose over the suggested scheme for modifying the financial basis of the Act with a view to preventing or minimising the disclosure of deficiencies at the first valuation, and thus of obviating the reduction of benefits.

It will be remembered that the Departmental Committee were charged with the duty of preparing a scheme which should not require additional moneys from Parliament, nor alter the present scale of contributions and benefits, but under which it should be possible to adjust the cost of the benefits to the contributions available. In these circumstances there was but one course open, and that was to apply the sinking fund to present needs rather than to future benefits. The suggested "raid upon the sinking fund" was combined, it is true, in the recommendations contained in their

### NATIONAL INSURANCE DEVELOPMENTS (*continued*).

Interim Report with the setting up of various contingency and equalisation funds, and, moreover, it was distinctly stipulated as an integral part of the scheme that it should be brought under consideration with a view to revision when the Act had been ten years in force, at which time it was anticipated that the accumulated experience would furnish a reliable basis for the permanent reconstruction of the financial scheme. Nevertheless, the net effect of the proposals was to defer for several years the time when the generation now entering into insurance at the youngest ages would secure any benefit from the Government grant towards the cost of their insurance.

### DEFERMENT OF ULTIMATE REFORM.

It was this deferment of ultimate reform to a more convenient season which the Commission of Investigation was called into being to resist. From the outset the plea has been for additional money to be provided by the Treasury to put matters right if they are found to be wrong—and there is a good deal that might be said in favour of this contention. As, however, no valuations have yet been made no one yet knows exactly to what extent, if at all, the approved societies would show deficiencies at their first valuations. It is certainly probable that the information available to the actuaries at the offices of the Insurance Commissioners, and through them to the official Departmental Committee, placed the Committee in a much stronger position in regard to facts than could possibly be the case with an unofficial investigation. Moreover, the Departmental Committee had the advantage of the chairmanship of Sir Gerald Ryan as well as the services of Mr. Ernest Woods and Sir Alfred Watson, the actuary to the Insurance Commissioners. Actuaries are naturally cautious—it is essential that they should be—and with such weighty professional guidance it is unthinkable that the scheme proposed by the Departmental Committee would not have been found to operate with satisfaction within the limits assigned. And in the absence of any truly reliable data it was undoubtedly a prudent recommendation that the final settlement should be deferred until such time as it would be possible to command full approval for the scheme on the ground of actual experience.

### THE CONCLUSIONS SUMMARISED.

The Commission of Investigation, however, appeared to have considered that the consensus of such weighty professional opinion could only mean that something was at present radically wrong with the finance. Having started with this bias the Report now issued naturally gives greatest prominence to this aspect of the matter, and the conclusions under this heading may be summarised as follows, namely:—

1. That no alteration should be made until the facts are disclosed.
2. That the recommendations of the Departmental Committee would undermine the status of approved societies.
3. That the resort to the sinking fund is unsound.
4. That proposals which might have the effect of destroying small societies are unwarrantable.
5. That sufficent regard has not been paid to disablement benefit, and that if the Treasury grant is to be available it should be utilised for the amelioration of the difficulties which exist, irrespective of this benefit.
6. That the depreciation in the value of the assets invested through the National Debt Commissioners should fall upon public funds.
7. That no portion of the cost of the War should be thrown upon approved societies.
8. That any scheme of relief should deal with the finances pertaining to each sex separately.
9. That average reserves and contributions do not meet the circumstances of approved societies constituted as at present.
10. That conditions are rapidly and constantly changing, and that any amendments should be treated as being outside the sphere of partisan conflict.

### IS A TREASURY GRANT POSSIBLE?

It will be observed that the conclusions are in the main merely criticisms of the Report of the Departmental Committee, and that no direct recommendation is made as to a Treasury grant. But without such a grant the position of the societies which have taken more than their due proportion of hazardous risks would remain unrelieved. Hence the plea is for a special Treasury grant, and the question therefore resolves itself into this—Is such a grant likely to be forthcoming? If the answer is that the heavy war expenditure makes such a grant impossible at the present time, then there is really no alternative to a scheme more or less on the lines of the Report of the Departmental Committee. The decision as to making a further grant must remain with the Government, and no good purpose would be served in pressing unduly for further State assistance at the present time.

There is one consideration raised by the publication of this Report to which, in conclusion, we must refer. It is this: Whatever may be the merits or demerits of the scheme of financial reform proposed by the Departmental Committee, there can be no two opinions as to the desirability of carrying into effect the simplifications of administrative machinery, proposed in their further Report. The reforms are required urgently, and new legislation is required to enable them to be carried out. But this legislation is unobtainable unless the Bill can be given the character of an uncontentious measure. This was very strongly urged by Sir Edwin Cornwall (the new Insurance Minister) at a recent luncheon given in his honour. He then emphasised the importance of securing harmony between all the various National Insurance interests. It would, therefore, be most unfortunate if the publication of the Report of the Commission of Investigation should be taken as an occasion for stirring up strife, and we support the proposals of the Departmental Committee for deferring—but not postponing indefinitely—the time when the whole scheme can be reviewed under more favourable conditions.

